# Characterization of human living myocardial slices culture-induced adaptations: a translational perspective

**DOI:** 10.1016/j.jmccpl.2025.100465

**Published:** 2025-06-17

**Authors:** Jort S.A. van der Geest, Ernest Diez Benavente, Willem B. van Ham, Pieter A. Doevendans, Linda W. van Laake, Teun P. de Boer, Vasco Sampaio-Pinto, Joost P.G. Sluijter

**Affiliations:** a Department of Cardiology and Experimental Cardiology Laboratory, Division of Heart & Lungs, University Medical Centre Utrecht, Utrecht, the Netherlands; b Regenerative Medicine Centre Utrecht, Circulatory Health Research Center, University Utrecht, University Medical Centre Utrecht, Utrecht, the Netherlands; c Department of Medical Physiology, Division of Heart & Lungs, University Medical Centre Utrecht, Utrecht, the Netherlands; d Netherlands Heart Institute (NLHI), Utrecht, the Netherlands; e Central Military Hospital (CMH), Utrecht, the Netherlands

**Keywords:** Living myocardial slices, Biomimetic culture, Remodeling, Translational research, Drug development, Cardiac function

## Abstract

Heart failure involves complex pathophysiological processes, best studied in multicellular human cardiac tissues that reflect the native cellular composition and microenvironment. However, maintaining primary cells and tissues in culture for extended periods remains challenging. Developing robust human cardiac models is critical for advancing preclinical research and bridging the gap to clinical applications. This study aims to characterize adaptations occurring in human living myocardial slices (LMS) during *ex vivo* culture.

During culture, LMS demonstrated progressive enhancements in contractile function including stronger force generation, reduced diastolic tension, and faster contraction-relaxation kinetics. However, excitability and force-frequency response decreased over the same period. Cultured LMS showed enhanced calcium handling, including increased ability to follow pacing, higher amplitude, and faster, more stable calcium re-uptake. Structurally, LMS displayed no changes in sarcomeres, cell-cell connections, or mitochondria, despite gene expression changes in cytoskeletal and extracellular matrix-related pathways. Transcriptomic analysis revealed metabolic activation with upregulation of metabolism-related pathways. Interestingly, LMS exhibited increased expression of genes associated with early cardiac development after the culture period.

LMS provide a powerful translational model for cardiovascular research, enabling the evaluation of novel therapies and fundamental studies. However, culture-induced adaptations must be carefully considered when interpreting results to ensure physiological and disease relevance.

## Highlights

• LMS improve functionally but lose excitability during the first 10 days of culture.
• Transcriptomic culture-adaptation of LMS varies across cardiac cell types.
• LMS retain structure despite showing ECM and cytoskeletal transcriptomic changes.
• LMS undergo metabolic remodeling in response to culture conditions.
• LMS reactivate developmental cardiac gene programs under culture conditions.

## Graphical abstract

**Figure:**
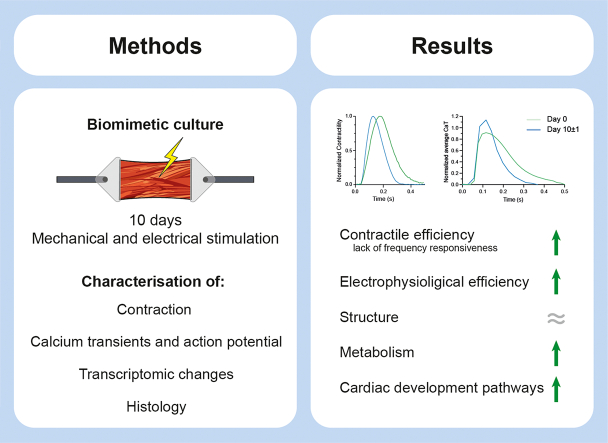

## 1 Introduction

Heart failure (HF) is a complex pathophysiological disease with a rising incidence driven by improved early-stage therapies that increase life expectancy but result in more patients progressing into end-stage HF requiring advanced or curative interventions [1,2]. However, in 2023, the number of new cardiovascular drugs approved by the FDA was lower than the average from the previous 5 years [3], which was already notoriously low [4]. The lack of accurate recapitulation of human (patho)physiology by preclinical models hampers the implementation of novel therapies in the clinic. Attempting to overcome this hurdle, the Modernization Act 2.0 by the FDA has marked a regulatory paradigm shift, allowing exclusively non-animal methodologies to establish drug safety and efficacy.

Currently, the main focus on understanding HF and developing therapies has shifted toward the use of human-based models, such as human-induced pluripotent stem cells-derived cells (hiPSCs), including cardiomyocytes (hiPSC-CMs), as well as engineered heart tissues (EHTs). While the high throughput capacity of these models facilitates the discovery of novel therapeutics, these traditional models often face limitations in recapitulating the intricate cellular interactions, extracellular matrix (ECM), and physiological properties characteristic of the native myocardium, especially in replicating years of pathological remodeling. As a result, there is a critical gap in translating findings from animal-based and *in vitro* systems to clinical applications, often referred to as the “valley of death” of drug development [5]. Developing more reliable and physiologically-relevant research models is essential to advance our understanding of cardiovascular diseases and drive the testing of the efficacy and safety of new treatment strategies.

One promising approach to bridge this gap is using human living myocardial slices (LMS). These 300 μm-thick slices of the human heart can maintain the complex tissue architecture and cellular composition of the native myocardium [6,7]. Moreover, if LMS are taken from a diseased heart, they contain the biological and mechanical cues associated with years of pathological remodeling. The advent of biomimetic culture systems, which mimic the *in vivo* myocardial microenvironment through electrical and mechanical stimulation, has challenged the dogma that cardiac *in vitro* models inevitably degrade in culture [8,9]. By allowing the culturing of LMS for months [6], it is now possible to conduct a human preclinical assessment of chronic therapeutic interventions. Previous studies have explored culture-induced adaptations of LMS, primarily focusing on characterizing biomimetic culture systems. However, while these studies have addressed aspects of functional adaptation, a detailed biochemical and functional comparison between fresh and cultured cardiac tissue over time is still lacking [6,7,10]. LMS have been shown to reach a stable state after 2 to 3 weeks of culture, with the most prominent changes occurring shortly after culture initiation. Therefore, the focus of these studies has largely been on the later stages of LMS culture [6,11]. In contrast, our study emphasizes the initial phase of LMS culture to provide a deeper, translational perspective. As the use of LMS culture for testing novel therapeutics and exploring the fundamental mechanisms of cardiac (patho)physiology grows, understanding temporal adaptation is essential.

Here, we provide a longitudinal and multidimensional characterization of fresh LMS and their cultured counterparts to investigate how LMS adapt to culture conditions over time. This evaluation is essential for determining the utility of LMS as a model system for translating preclinical research, particularly in assessing the efficacy and safety of novel cardiovascular therapies.

## 2 Methods

A detailed description of the methods is available in the supplemental material.

### 2.1 Study approval

Experiments were approved by the local medical ethics board and written informed consent is provided by all patients for myocardial biopsies UCC-UNRAVEL #12-387 [12].

### 2.2 Statistical analysis

Statistical analyses were conducted using R (v4.2.2) in R-studio and GraphPad Prism 10.4.1. Patient characteristics from which the LMS were obtained, were generated using tableone package. For repeated measurements, a mixed-effect modeling approach was used, followed by *post hoc* analysis employing Tukey's multiple comparisons method. Values are presented as mean ± SEM. The statistical significance level chosen for all statistical tests was *p* < 0.05.

## 3 Results

### 3.1 Contractile remodeling of LMS during culture

In this longitudinal study, we characterized patient-derived LMS over 10 days of culture and evaluated how well cultured LMS recapitulate the characteristics of the patient's fresh uncultured cardiac tissue. The LMS were generated from freshly obtained left ventricular myocardial biopsies, and the baseline characteristics of the patients from whom the LMS were derived are presented in Table 1.

**Table 1:** Baseline characteristics of patients' biopsies for LMS generation.

N	11
Age (years, median [IQR])	52 [51, 63]
Sex = M, n (%)	9 (82 %)
Pre-existing LVAD = Y, n (%)	8 (73 %)
Etiology, n (%)	
ACM	1 (9 %)
CTRCD	1 (9 %)
HCM^a^	1 (9 %)
DCM	6 (55 %)
ICM	2 (18 %)
Tissue originating procedure, n (%)	
Heart transplantation	9 (82 %)
LVAD	1 (9 %)
Post-mortem	1 (9 %)
Class 4/5 mutations, n (%)	
*LMNA*	1 (9 %)
*PLN*	2 (18 %)
*TTN*	3 (27 %)
None	5 (46 %)
Comorbidities, n (%)	
Diabetes Mellitus	1 (9 %)
Clinically relevant ventricular tachycardia	4 (36 %)
Atrial fibrillation	3 (27 %)
Medication, n (%)	
ACEi/ARB/ARNI	6 (55 %)
Beta-blocker	3 (27 %)
Calcium antagonist	5 (46 %)
Class 3 anti-arrhythmics	8 (73 %)
Glycoside	1 (9 %)
MRA	6 (55 %)
SGLT2-inhibitor	2 (18 %)

a HCM in a dilating phase. ACEi, angiotensin-converting enzyme inhibitors; ACM, arrhythmogenic cardiomyopathy, ARB, angiotensin receptor blocker; ARNI, angiotensin receptor/neprilysin inhibitor; CTRCD, cancer therapy-related cardiac dysfunction; DCM, dilated cardiomyopathy; HCM, hypertrophic cardiomyopathy; ICM, ischemic cardiomyopathy; *PLN*, phospholamban; LVAD, left ventricular assist device; *LMNA*, Lamin A/C; M, male; MRA, mineralocorticoid receptor antagonist; *TTN*, Titin; SGLT2, sodium-glucose cotransporter 2.

**Fig. 1:**
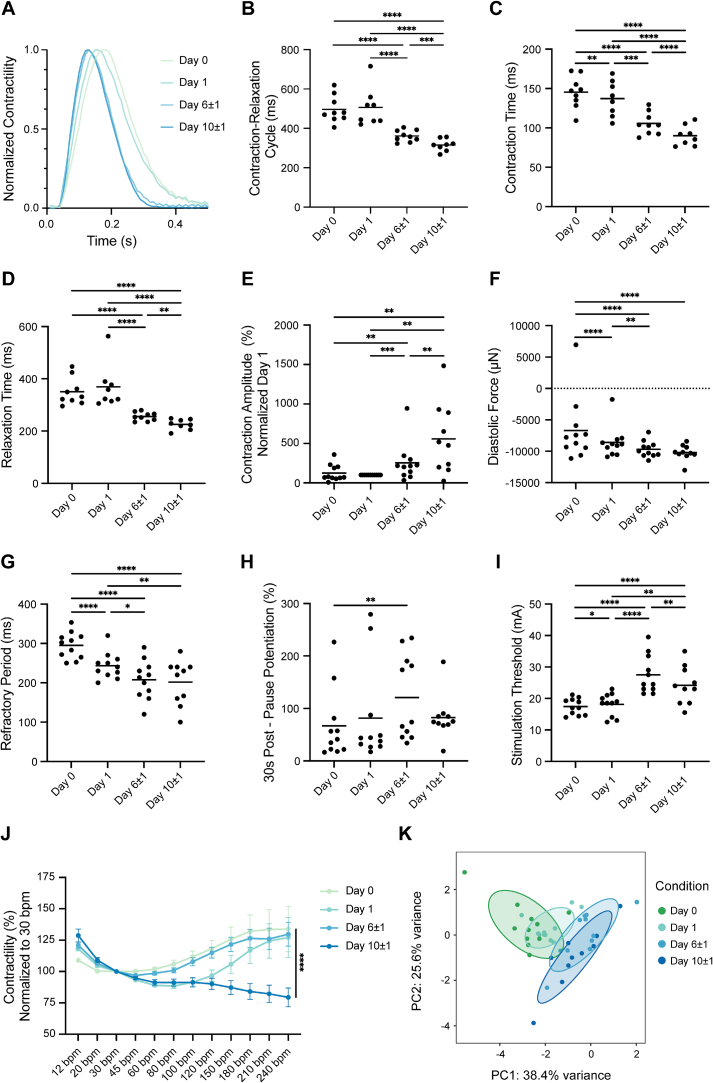
Functional remodeling of LMS during culture. A) Representative contraction transient at different days of culture. Cultured LMS show a faster contraction-relaxation cycle (B), attributed to a reduction in both the contraction (C) and relaxation times (D) (A–D: Day 0 *N* = 9, *n* = 19; Day 1 *N* = 8, *n* = 16; Day 6 ± 1 N = 9, n = 19; N = 8, *n* = 17). Contraction force increases during culture (E), while diastolic tension decreases (F). The refractory period (G) decreases, while the post-pause potentiation (H) and stimulation threshold (I) increase. J) Cultured LMS lose the ability to develop more force when subjected to higher pacing frequencies while being more effective at lower frequencies (Tukey's multiple comparisons are provided in Supplemental Fig. S1D); (E–J: Day 0 N = 11, *n* = 126; Day 1 *N* = 11, *n* = 61; Day 6 ± 1 N = 11, *n* = 49; *N* = 10, *n* = 22). K) PCA displaying changes between LMS exposed to culture for 0, 1, 6 ± 1 and 10 ± 1 days based on five functional parameters (stimulation threshold, post-pause potentiation after a 30-second pause, contractile force, diastolic force, and refractory period) (N = 11). Ellipses indicate the standard deviation. In all experiments, N represents the number of unique patients from whom the LMS were derived, and n represents the number of LMS. Statistical analyses were performed using a mixed-effects model accounting for repeated measures within subjects, followed by Tukey's multiple comparisons test. Data are presented as mean ± SEM. Statistical significance was defined as *p* < 0.05.

As illustrated in Fig. 1A, the representative contraction transients progressively shortened over the first 10 days of culture. The complete contraction-relaxation cycle duration decreased (Fig. 1B), due to a faster contraction (Fig. 1C) and relaxation time (Fig. 1D). In addition, as culture time increased, LMS displayed an enhanced contraction force (Fig. 1E; absolute forces are provided in Fig. S1A) and a subtle reduction in diastolic force (Fig. 1F). Overall, the contraction and relaxation became more efficient, reflecting an improved contractile adaptation.

In line with this, LMS exhibited a reduced refractory period (Fig. 1G) and increased post-pause potentiation, particularly evident at longer pause intervals (3 s, 12 s, 30 s) (Fig. 1H and Supplemental Fig. S1B and C). This indicates improved ion homeostasis and reserves underlying the contractile adaptation. Conversely, the current required for LMS contraction (*i.e.* stimulation threshold) increased during culture (Fig. 1I). Additionally, the force-frequency relationship showed that at Day 10 ± 1, LMS were no longer able to increase the contractile force at higher pacing frequencies while force development was stronger at lower pacing rates (Fig. 1J). To summarize the functional remodeling during culture, a principal component analysis (PCA) was performed to evaluate changes in stimulation threshold, post-pause potentiation after a 30-second pause, contractile force, diastolic force, and refractory period. A temporal gradient is presented in the PCA in Fig. 1K, confirming the functional adaptation of LMS during culture.

### 3.2 LMS undergo cellular and molecular remodeling upon culture

**Fig. 2:**
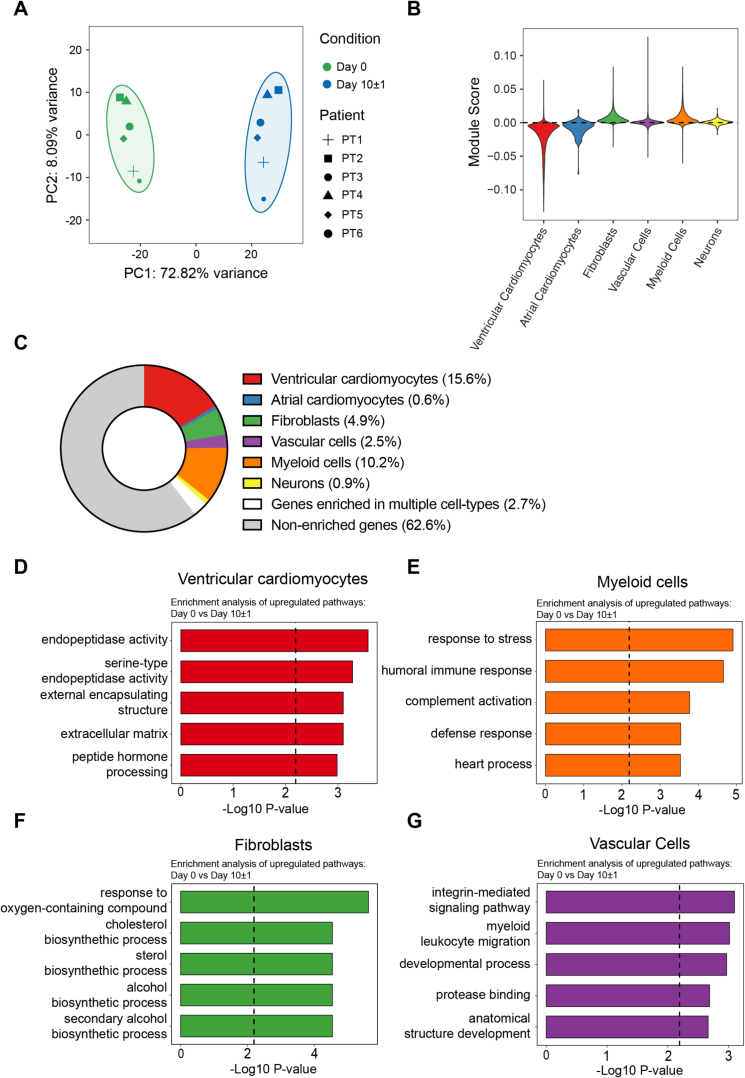
LMS undergo cellular and molecular changes upon culture. A) Principal component analysis plot based on the 500-most differentially expressed genes in day 0 *vs* 10 ± 1 days cultured LMS. Ellipses indicate the standard deviation. B) Module score of the differentially upregulated genes between day 0 and day 10 ± 1. C) Mapping upregulated genes to cell markers specific to the different cell types in the heart (ventricular and atrial cardiomyocytes, fibroblasts, vascular cells, myeloid cells, and neurons). Pathway enrichment analysis of ventricular cardiomyocytes (D), myeloid cells (E), fibroblast (F), and vascular cells (G) on day 0 *versus* day 10 ± 1 cultured LMS. For all panels, *N* = 6 unique patients and *n* = 6 LMS were used.

The transcriptomic profile of LMS, assessed by bulk RNA sequencing, differed between day 0 and day 10 ± 1 (Fig. 2A). A key advantage of LMS is preserving the multicellular composition of the patient's heart. To assess the adaptation of different cell types in the heart, the differentially upregulated genes were mapped to a publicly available single-cell RNA sequencing dataset of the left ventricle [13]. Module score analysis (Fig. 2B) shows the magnitude of gene expression changes across different cell types. Ventricular and atrial cardiomyocyte-associated genes exhibited negative mean module scores (−0.0193 and −0.00877, respectively), indicating a lower enrichment of upregulated genes in cardiomyocytes during culture. In contrast, myeloid cells (mean module score = 0.00697) and fibroblasts (mean module score = 0.00667) exhibited the strongest enrichment of upregulated genes, while vascular cells (mean = 0.000358) and neurons (mean = 0.000636) contributed minimally to the upregulated gene pool.

Out of the 4132 upregulated genes identified during culture, 1764 were matched to the single-cell transcriptomic data from the left ventricle. Of these, 15.6 % were enriched in ventricular cardiomyocytes, 0.6 % in atrial cardiomyocytes, 4.9 % in fibroblasts, 2.5 % in vascular cells, 10.2 % in myeloid cells, and 0.9 % in neurons. These findings suggest an active involvement of these cell types in the functional remodeling of LMS during culture (Fig. 2C and Supplementary Fig. S2). Pathway enrichment analysis for each cell type revealed the key functions altered in the different cell types: changes in ventricular cardiomyocytes were linked to peptide hormone processing and ECM interactions (Fig. 2D). Myeloid cells were enriched in immune-related responses and stress adaptation pathways (Fig. 2E). Fibroblasts exhibited shifts in metabolic processes (Fig. 2F), while vascular cells upregulated pathways related to development, ECM interactions, and integrin signaling, supporting vascular remodeling. These findings demonstrate that different cell types within LMS responded distinctly *via* molecular pathways, contributing to the observed functional remodeling during culture and highlighting the complexity and adaptability of this multicellular system.

### 3.3 Electrophysiological remodeling of LMS during culture

**Fig. 3:**
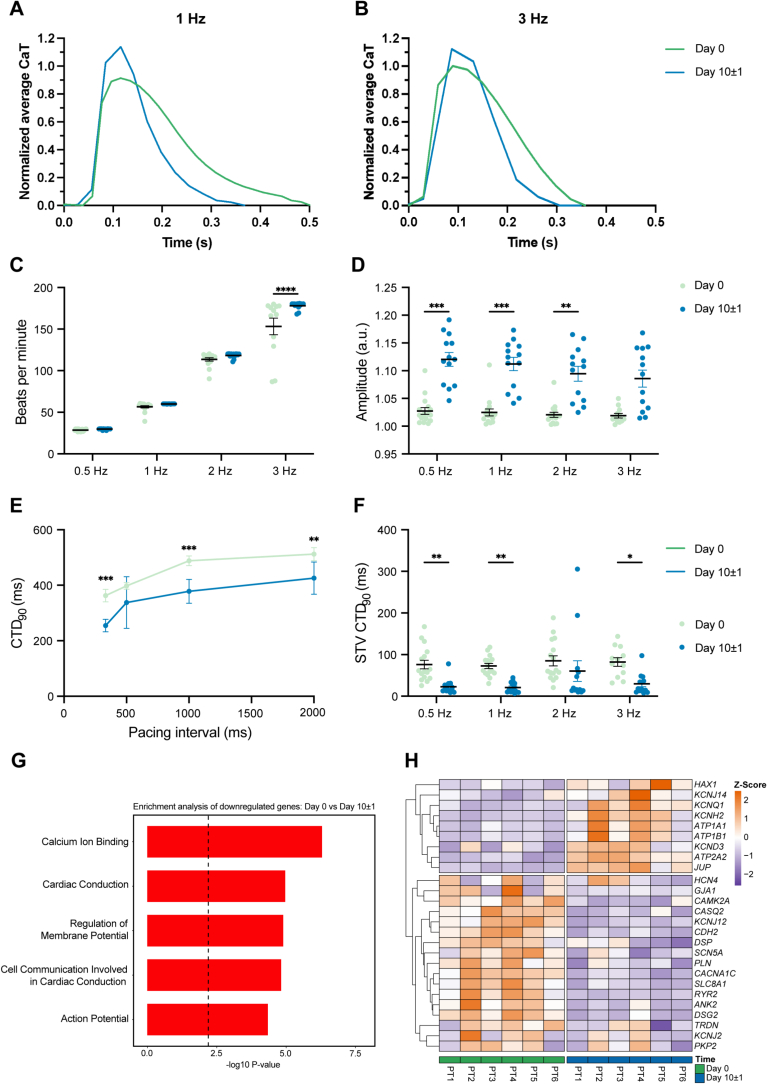
Electrophysiological remodeling of LMS during culture. Average calcium transient at 1 Hz (A) and 3 Hz (B) at day 0 and day 10 ± 1 of culture. At day 10 ± 1 of culture, LMS show an increased ability to follow pacing at higher frequencies (C), and increased calcium amplitude (D). Calcium transient decay time (CTD_90_) (E) is shorter and has a decreased short-term variability (STV) (F) at day 10 ± 1 of culture (C–F: day 0 *N* = 6, *n* = 16, day 10 ± 1 N = 6, *n* = 13). Pathway enrichment analysis on electrophysiological (G) and contractility (H) pathways on day 0 *versus* day 10 ± 1 cultured LMS (N = 6, *n* = 6). I) Heatmap representation of differentially expressed genes of key players associated with cardiac electrophysiology (*N* = 6, *n* = 6). Where N represents the number of patients of which LMS were derived and n indicates the individual LMS. Statistical analyses were performed using a mixed-effects model accounting for repeated measures within subjects, followed by Tukey's multiple comparisons test. Data are presented as mean ± SEM. Statistical significance was defined as *p* < 0.05.

To assess electrophysiological changes during culture, we optically analyzed calcium handling in freshly prepared LMS and after 10 ± 1 days of culture (Fig. 3A and B). Cultured LMS demonstrated an improved ability to follow pacing up to 3 Hz, whereas fresh LMS were unable to consistently follow pacing (Fig. 3C). Calcium transient amplitude increased in cultured LMS (Fig. 3D), consistent with the enhanced post-pause potentiation (Fig. 1H and Supplemental Fig. S1B and C). Shorter refractory periods in cultured LMS (Fig. 2G) aligned with decreased calcium transient duration (CTD_90_) and lower short-term variability (STV) of CTD_90_ (Fig. 3A, B, E, F). Transcriptomic analysis revealed a significant downregulation of electrophysiological and contraction-related pathways in cultured LMS (Fig. 3G and H), influenced by altered expression of key ion channels (Fig. 3I). Overall, adaptation to culture conditions resulted in a more stable and efficient calcium cycling in LMS.

### 3.4 Structural remodeling of LMS during culture

**Fig. 4:**
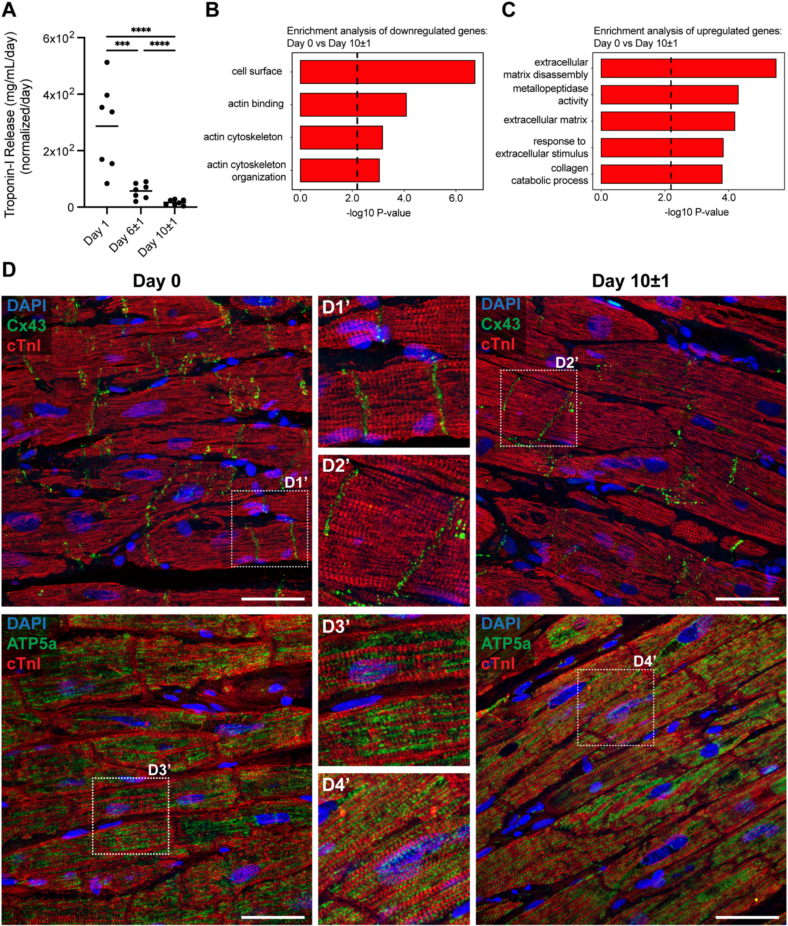
Structural remodeling of LMS during culture. A) Troponin-I release by LMS to the medium (*N* = 7, *n* = 15). Pathway enrichment analysis on cytoskeletal (B) and extracellular matrix (C) pathways on day 0 *versus* day 10 ± 1 cultured LMS (C & D: *N* = 6, *n* = 6). D) Representative confocal images of cardiac troponin-I (cTnI), gap junction alpha-1protein (Cx43), and mitochondrial ATP synthase F1 subunit alpha (ATP5a) immunolabelling of LMS at day 0 and day 10 ± 1 of culture (*N* = 3, *n* = 3), scale bars = 50 μm. N indicates the number of unique patients from whom the LMS were derived, n represents the individual LMS. Statistical analyses were performed using a mixed-effects model accounting for repeated measures within subjects, followed by Tukey's *post hoc* test. Significance was set at p < 0.05.

To determine whether there is ongoing damage and cardiomyocyte loss during culture, Troponin-I release in the conditioned media was measured. Troponin-I levels were high (mean = 277.8 mg/mL/day) 1 day after LMS production but rapidly diminished (mean day 10 ± 1 = 16.1 mg/mL/day) (Fig. 4A), indicating that initial stress or damage from slicing was not sustained during culture. At the RNA level, pathways related to cytoskeletal structure were downregulated, while pathways associated with ECM remodeling were upregulated (Fig. 4B and C) in cultured LMS, reflecting molecular-level adaptations to the culture environment. However, histological validation showed that cultured LMS retained regular sarcomere striation (Troponin I), well-defined gap junctions (Cx43), and intact mitochondrial network (ATP5a), all indistinguishable from their fresh (day 0) counterparts (Fig. 4D). These findings indicate that structural integrity and organization are maintained in cultured LMS, with no evidence of ongoing damage to the myocardial cells, despite transcriptomic changes.

### 3.5 Metabolic activation of LMS during culture

**Fig. 5:**
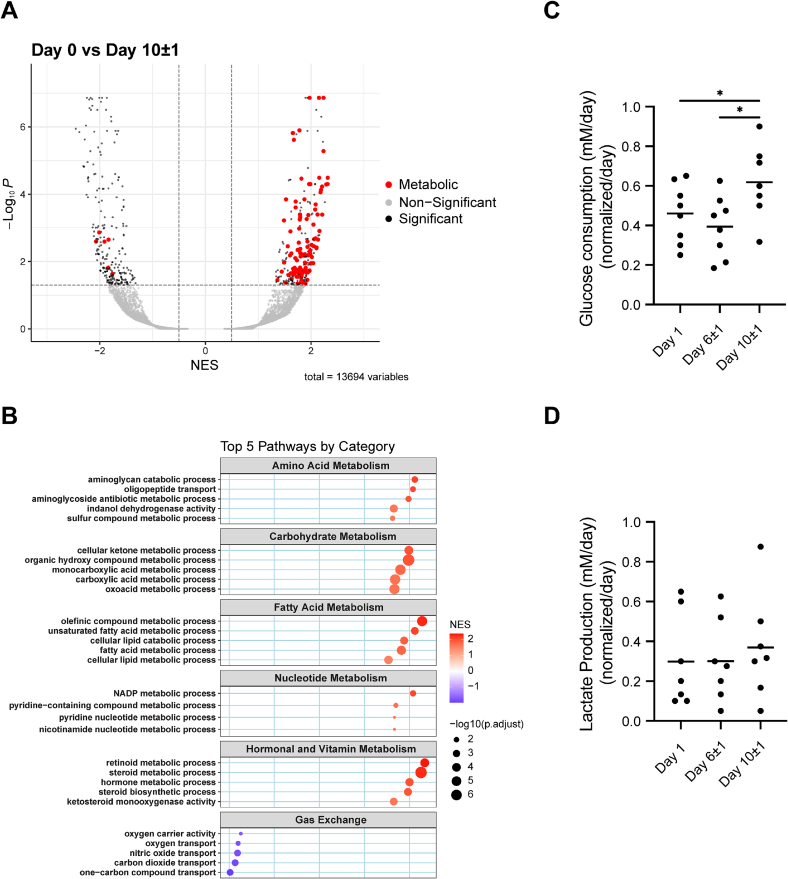
Metabolic remodeling of LMS during culture. A) Volcano plot of differential pathways associated with metabolism. B) Pathway enrichment is subdivided into the four main metabolic pathways: amino acid, carbohydrate, fatty acid, and nucleotide metabolism, and pathways related to hormones and vitamins and gas exchange (A–B: N = 6, n = 6). Glucose consumption (C) and lactate production (D) were measured in the conditioned media of LMS (C–D: *N* = 8, *n* = 18). N refers to individual patient donors, and n indicates the number of LMS analyzed. Statistical analyses were performed using a mixed-effects model accounting for repeated measures within subjects with Tukey's multiple comparisons test and statistical significance was defined as p < 0.05.

Following previous observations, we characterized the metabolic remodeling that occurred during culture. Notably, on a transcriptomic level, metabolism-related pathways formed the majority of the upregulated pathways during culture (Fig. 5A), while the downregulated pathways were associated with gas exchange. A detailed evaluation of specific metabolic pathways, including amino acid, carbohydrate, fatty acid, and nucleotide metabolism showed varying degrees of upregulation (Fig. 5B). We also detected a significant upregulation of pathways associated with hormonal and vitamin metabolism (Fig. 5B). In line with these transcriptomic changes, glucose consumption increased over time in culture from an average of 0.47 to 0.63 mM/day between day 1 and day 10, while assessment of lactate production levels showed a non-significant trend to increase from 0.29 to 0.37 mM/day (Fig. 5C and D). These findings show that LMS activate metabolic pathways during culture to adapt to the artificial environment and support the functional alterations.

### 3.6 Developmental shift in LMS during culture

**Fig. 6:**
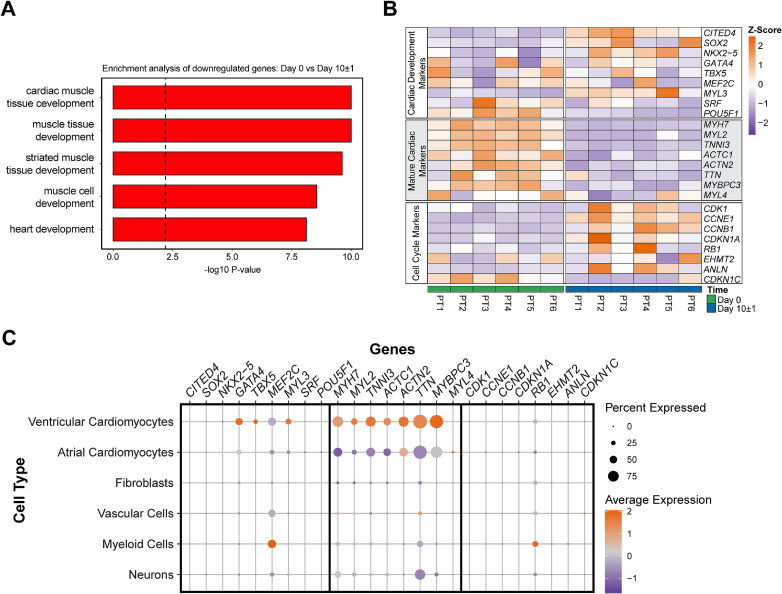
Developmental shift in LMS during culture. A) Pathway enrichment analysis of developmental pathways on day 0 *versus* day 10 ± 1 cultured LMS (N = 6 unique patients, n = 6 individual LMS). B) Heatmap representation of differentially expressed genes associated with cardiac development markers, cardiac maturation markers, and cell cycle markers in LMS at day 0 and day 10 ± 1 of culture (N = 6 unique patients, n = 6 individual LMS). C) Dot plot of the expression of markers associated with cardiac development markers, cardiac maturation markers, and cell cycle markers in the different evaluated cell types.

Interestingly, cardiac development-related pathways were strongly represented among the differentially expressed pathways during culture. The top five pathways related to development are shown in Fig. 6A. To gain insights into this observation, we created a heatmap of genes associated with 3 categories: 1) cardiac development markers, 2) cardiac maturation markers, and 3) cell cycle markers. After 10 ± 1 days of culture, the expression of cardiac maturation markers was significantly reduced, while cardiac development markers, as well as cell cycle markers, were upregulated (Fig. 6B). To attribute these patterns to specific cell types and assess the differentiation state of the LMS in culture relative to freshly isolated cardiac tissue, we compared our data to publicly available single-cell data from a healthy left ventricle [13]. This analysis revealed that cardiac development markers and cardiac maturation markers are primarily expressed in ventricular cardiomyocytes, while cell cycle markers are not specific to any cell type (Fig. 6C). Together, these findings indicate that LMS undergo a developmental shift during culture, marked by a partial reactivation of early cardiac gene programs.

## 4 Discussion

### 4.1 LMS undergo remodeling upon culture

LMS are increasingly used as an *ex vivo* platform to model human cardiac (patho)physiology and evaluate novel therapeutics. However, it is crucial to understand how LMS adapt to culture conditions over time and whether they continue to represent the native myocardial tissue. In this study, we conducted a multidimensional analysis to investigate the culture-induced alterations in LMS, focusing on their molecular, functional, structural, and metabolic remodeling. We demonstrated that LMS undergo extensive remodeling, with key changes in their function and molecular profile. Functionally, LMS exhibited enhanced contractile properties, including stronger contraction force, reduced diastolic tension, and faster contraction and relaxation kinetics. However, LMS became less responsive to electrical stimulation as evidenced by an increase in stimulation threshold and decreased force development at higher pacing rates. Mechanistically, cultured LMS exhibited improved calcium handling with an increased ability to follow pacing, increased amplitude, and faster and more stable calcium re-uptake. Structurally, LMS showed no distinguishable changes in sarcomeres, cell-cell connections, and mitochondria, despite the downregulation of cytoskeletal-associated genes and upregulation of ECM-related genes, indicating that structural integrity was maintained during culture. In addition, transcriptomic analysis revealed a significant metabolic activation, with upregulated metabolism pathways, corresponding to an increased ability of LMS to utilize glucose over the culture period.

### 4.2 Transcriptional phenotypic shifts

The bulk transcriptomic analysis conducted herein precluded the evaluation of cellular composition. Nevertheless, alterations in cellular phenotype were detected *via* a single-cell-informed mapping approach with different cell types within the LMS responding distinctly to culture. Cardiomyocytes exhibited a downregulation of pathways linked to contractile function and electrophysiological properties, which contrasts with the observed functional improvements, such as a more efficient contraction and calcium handling. In contrast, fibroblasts and myeloid cells exhibited an upregulation of pathways related to metabolism and immune response, respectively, after culture. Previous studies have shown that physiologic mechanical loading of LMS inhibits fibroblast proliferation [8,9]. This suggests that the changes observed herein are the result of a shift in the functional state of the fibroblasts rather than a mere overgrowth as seen in unloaded culture conditions. Similarly, the culture environment is devoid of circulating immune cells and pro-inflammatory cytokines and chemokines, in contrast to the *in vivo* HF setting. Consequently, we found LMS's myeloid cells to upregulate pathways related to ECM interactions, immune responses, and stress adaptation pathways upon culture.

### 4.3 Upregulation of developmental pathways in ventricular cardiomyocytes

Surprisingly, we observed a downregulation of cardiac maturation markers concomitant with an increased expression of cardiac development markers, suggesting a developmental shift of LMS's cardiomyocytes toward a more fetal cardiac stage. The reactivation of fetal gene programs has been shown upon unloading by a left ventricular-assist device (LVAD) [14,15]. This shift in gene expression has been associated with clinical improvement including reversal of pathological cardiomyocyte hypertrophy, enhancement of contractile function, and normalization of heart dimensions, such as the restoration of normal heart shape and reversal of dilatation. In acute and potentially reversible forms of HF, like myocarditis, peripartum cardiomyopathy, and drug-induced cardiomyopathy, LVADs can be used transiently to promote functional restoration, followed by explantation upon recovery [[16], [17], [18]]. The observed functional improvements in cultured LMS may reflect, at least partially, the reparative unloading seen in end-stage HF patients after LVAD implantation. We hypothesize that the physiological loading applied during LMS culture, resulting in a sarcomere length of ∼2.2 μm, constitutes a relative unloading compared to the chronically overloaded myocardium of end-stage heart failure patients, where a sarcomere length > 2.4 μm has been reported [7,8]. However, tissue mechanics across patient populations is highly variable and dependent on disease etiology, degree of fibrosis, and prior mechanical support, *e.g.* LVAD. Future studies should explore whether increasing mechanical load to more closely replicate the overloaded conditions could better retain end-stage HF features in culture.

### 4.4 Translational considerations for LMS culture

Culturing LMS in a biomimetic culture system aims to mimic the natural cardiac microenvironment to preserve the functional state of the myocardium. However, as our study shows, LMS adapt to the culture environment. Specifically, the reduced excitability and force development at higher pacing frequencies may reflect adaptations to the subphysiological pacing of 0.5 Hz. In contrast, the improved contractile and electrophysiological efficiency represents a form of phenotypic recovery in LMS derived from end-stage HF patients. This recovery could serve as a valuable model for investigating mechanisms underlying functional repair or regeneration, especially therapies that might synergize LVAD-induced reverse remodeling. Notably, the majority of cultured LMS in our study were derived from tissue previously exposed to LVAD implantation, indicating that an additional reverse remodeling can be induced.

Depending on the experimental goal, these culture adaptations can be either beneficial or undesirable. For drug testing, a less severe disease stage may be necessary to better model the effects of potential therapies before the condition becomes refractory. However, clinical trials often target end-stage patients, where the disease is resistant to treatment. Therefore, optimizing culture conditions is crucial to fully retain disease-specific features, ensuring that LMS can accurately reflect the relevant disease stage for the intended research. The clean and synthetic culture environment may not adequately capture the pro-inflammatory and high-stress environment of a failing heart, evident from the observed phenotypic switch in fibroblasts and myeloid cells. In line, LMS are currently exposed to a glucose-rich medium, which likely contributes to the upregulation of metabolic pathways and the observed shift toward glucose utilization. The absence of fatty acids may further accentuate this adaptation, limiting activation of β-oxidation pathways. Therefore, we hypothesize that to fully preserve the end-stage HF features, LMS might need to be cultivated under conditions that more closely mimic the pathological *in vivo* conditions. This includes mechanical overloading and mimicking the circulating milieu through the addition of lipid substrates and pro-inflammatory cytokines such as tumor necrosis factor-alpha (TNF-α), interleukin-1 beta (IL-1β), interferon-gamma (IFN-γ) or transforming growth factor-beta (TGF-β). Supportive of this hypothesis, it has been previously demonstrated that short-term overloading of LMS does lead to pathophysiological remodeling, as demonstrated by reduced contractile function and cardiomyocyte hypertrophy [7,19] and fibrotic remodeling [8]. Future work could explore whether introducing these pathological stimuli helps retain the heart's native diseased state. Additionally, subjecting LMS derived from healthy hearts, despite their limited availability, to the same physiological conditions could provide further insight into these findings.

Of note, the culture-induced adaptations observed in LMS must be considered during the experimental design (*e.g.* some sources of tissue might not be suitable for specific research questions) and always when translating findings to clinical applications. Despite these adaptations, LMS remain the best available option for replicating the human cardiac state and provide a valuable platform for advancing preclinical research, with great potential to accelerate drug development and enable more physiologically relevant disease modeling.

### 4.5 Study limitations

While the data presented here provides a detailed characterization of LMS remodeling during culture, several limitations should be noted. First, although cultured LMS became more efficient functionally, the transcriptomic data showed contrasting effects. These observed functional and molecular changes could potentially represent a compensatory recovery mechanism, as healthy myocardial tissue slices are not included. Future studies incorporating proteomic and post-translational modification analyses could provide more direct insights into the molecular mechanisms underlying the functional adaptations observed in LMS.

Despite the significant progress in LMS culture systems, they still represent a simplified version of the cardiac environment. Our findings provide insight into how optimizing culture conditions to better reflect *in vivo* conditions may mitigate these adaptations and enhance the relevance of the model. While the impact of these changes remains uncertain, they offer important clues for improving the LMS model and optimizing its use in preclinical research. Lastly, it is important to note that the observed functional variability likely reflects the broad biological diversity among patients, stemming from the difference in the underlying heart disease etiologies, comorbidities, and medication use. In our study, we used a predefined stretch protocol [7] to standardize sarcomere length across samples. While this approach is widely adopted, it does not account for potential differences in tissue mechanics and thus might result in variable sarcomere length between patient samples or disease states. Direct assessment of sarcomere length would provide a more accurate normalization across conditions and should be considered in future studies, as it may impact the degree of adaptation to culture. In addition, the LMS analyzed in this study were derived from 11 consecutively available donor tissues, the majority of which were from male patients. This gender imbalance could introduce sex-based biological differences that may influence both functional and transcriptomic outcomes. Future studies, with larger sample sizes and more balanced donor populations are needed to investigate potential sex-dependent effects in LMS responses to culture conditions. Despite these limitations, the phenotypical recovery of LMS in this study, highlights the remarkable plasticity and adaptability of the heart, even in a stage where HF is refractory to any treatment and transplantation is the only option. Such behavior warrants further in-depth investigation to understand how the specific disease context and patient-level factors influence the adaptive response observed in the LMS culture.

## 5 Conclusions

The synergy of LMS and biomimetic culture systems allows for bridging the gap between *in vitro* and *in vivo* experiments, offering a valuable platform for preclinical research. However, careful consideration of the model's needs and limitations is essential. While LMS undergo multidimensional remodeling, they remain the best available model for replicating the human cardiac state. Notably, even end-stage HF myocardium demonstrates a high degree of plasticity and responsiveness to environmental cues, which could potentially provide new insights into future curative therapeutic strategies. Chronic biomimetic culture of LMS models seems to model earlier stages of HF, providing a valuable platform for studying disease progression and testing interventions before end-stage HF. Nonetheless, optimizing culture conditions is still required to retain end-stage HF features. Ultimately, LMS hold great promise as a valuable *ex vivo* platform, with the potential to accelerate drug development and enable more physiologically relevant disease modeling.

## CRediT authorship contribution statement

**Jort S.A. van der Geest:** Writing – review & editing, Writing – original draft, Visualization, Validation, Resources, Project administration, Methodology, Investigation, Formal analysis, Data curation, Conceptualization. **Ernest Diez Benavente:** Writing – review & editing, Visualization, Supervision, Methodology, Formal analysis, Data curation. **Willem B. van Ham:** Writing – review & editing, Validation, Methodology, Formal analysis, Data curation, Conceptualization. **Pieter A. Doevendans:** Writing – review & editing, Supervision, Funding acquisition. **Linda W. van Laake:** Writing – review & editing, Supervision, Funding acquisition. **Teun P. de Boer:** Writing – review & editing, Supervision, Resources, Funding acquisition. **Vasco Sampaio-Pinto:** Writing – review & editing, Writing – original draft, Visualization, Validation, Supervision, Project administration, Methodology, Funding acquisition, Formal analysis, Conceptualization. **Joost P.G. Sluijter:** Writing – review & editing, Supervision, Project administration, Funding acquisition, Conceptualization.

## Funding

J.S.A.vd.G., P.A.D. and J.P.G.S. are supported by 10.13039/501100001826ZonMw Psider-Heart (10250022110004), 10.13039/501100003246NWO-TTP HARVEY (2021/TTW/01038252), the PLN foundation and ERA for Health Cardinnov (RESCUE-2024/KIC/01627794); Additionally J.P.G.S. is supported by 10.13039/100010661H2020-EVICARE (725229) of the European Research Council (ERC); L.W.v.L. is supported by the Dutch Heart Foundation: Dekker Senior Clinical Scientist 2019, grant agreement number 2019T056 and the Alliance Fund (UMCU, UU, TU/e); E.D.B. is funded by the 10.13039/501100000780European Union project European Research Council consolidator grant 866478 (UCARE) and the 10.13039/501100001674Leducq Foundation AtheroGEN; P.A.D. is supported by CUREPLaN Leducq (18CVD01) and 10.13039/501100000780EU Horizon 2022 grant GEREMY (grant agreement number 101080204). V.S.-P. is supported by a Netherlands Heart Institute postdoctoral fellowship.

## Declaration of competing interest

Joost Sluijter and Pieter Doevendans reports that financial support was provided by ZonMw Psider-Heart (10250022110004), NWO-TTP HARVEY (2021/TTW/01038252), the PLN foundation and ERA for Health Cardinnov (RESCUE- 2024/KIC/01627794). Joost Sluiter reports that financial support was provided by H2020-EVICARE (725229) of the European Research Council. Linda van Laake reports financial support was provided by Dutch Heart Foundation Dekker Senior Clinical Scientist 2019 (2019T056). Ernest Dieze Benavente reports financial support was provided by 10.13039/100010663European Research Council (866478). Pieter Doevendans reports financial support was provided by Europe Horizon 2022 grant GEREMY (101080204). Vasco Sampaio Pinto reports financial support was provided by the Netherlands Heart Institute. If there are other authors, they declare that they have no known competing financial interests or personal relationships that could have appeared to influence the work reported in this paper.

## Data Availability

RNA sequencing data used in this study is available upon reasonable request.

## Appendix A Supplementary data

Supplemental Table 1 Upregulated pathways. Supplemental Table 1

Supplemental Table 2 Downregulated pathways. Supplemental Table 2

Supplemental Table 3 Upregulated pathways Ven cardiomyocytes. Supplemental Table 3

Supplemental Table 4 Upregulated pathways myeloid cells. Supplemental Table 4

Supplemental Table 5 Upregulated pathway fibroblasts. Supplemental Table 5

Supplemental Table 6 Upregulated pathways vascular cells. Supplemental Table 6

Supplementary material Image 1
