# Supplementary material for: Characterization of human living myocardial slices culture-induced adaptations: a translational perspective
Source: J Mol Cell Cardiol Plus. 2025 Jun 17;13:100465. doi: 10.1016/j.jmccpl.2025.100465 (PMC12212253; doi:10.1016/j.jmccpl.2025.100465)
Supplement: Supplementary file 7 — Supplementary material [file mmc7.docx]

**Supplemental Appendix**

**Supplemental methods**

**LMS preparation**

LMS were prepared from 11 consecutive surgical procedures and cultured as in previously published work [1] with slight modifications. In brief, fresh cylindrical myocardial biopsy (radius 2 cm) were obtained from the left ventricle of patients with end-stage HF undergoing heart transplantation or immediate post-mortem examination or apical biopsy during LVAD implantation. The myocardial biopsies were transported on an ice-cold modified Tyrode’s solution (30 mM BDM, 0.9 mM CaCl_2_, 10 mM glucose, 10 mM HEPES, 9 mM KCl, 1 mM MgCl_2_ and 140 mM NaCl at a pH of 7.4). A summary of patient characteristics is given in Table 1.

Each myocardial biopsy was mounted with the epicardial side facing down and cut into 300 μm-thick LMS using a high-precision vibratome (7000 smz-2, Campden Instruments Ltd, Gillingham, UK). The vibratome was set to a z-axis error of <1.0 µm, a frequency of 80 Hz, 2 mm amplitude, and an advance speed of 0.03 mm/s. Slices were trimmed to approximately 7 mm x 7 mm and mounted perpendicular to the myofibril direction using 0.1 mm-thick polyester copier clear triangles and histoacryl glue (B. Braun, Melsungen AG, Germany). In biomimetic culture chambers (InVitroSys, München, Germany), LMS were continuously electrically paced (3 ms biphasic 50 mA pulses, at 0.5 Hz), stretched to a physiological stretch of 19.6% [2] and rocked at 60 rpm. The force of muscle contraction was continuously derived from the displacement of the magnetic tip of the spring wire slices are hooked to. More information on the design and validation of biomimetic cultivation chambers, can be found in [1] and in InVitroSys website. The LMS were cultured in medium 199 supplemented with 1% ITS, 0.2% primocin, 2.15 nM triiodothyronine (T3), 100 nM dexamethasone, 4 nM adrenaline, 4 nM noradrenaline, 50 μM 2-mercaptoethanol, and 20 μg/mL ascorbic acid (all from Sigma-Aldrich, Amsterdam, Netherlands). Media was changed every 48 to 72 hours and conditioned media was collected for analysis. A pacing rate of 0.5 Hz (50 mA, biphasic, 3 ms) was employed during culture and between series of automatic stimulation protocols, enabling the quantification of contractile force and kinetics. Each day at 1:00 AM, a custom-made stimulation protocol was run to determine the stimulation threshold, refractory period, force-frequency relationship and, post-pause potentiation. To determine the stimulation threshold, stimulus current was set at 95 mA and reduced by 3 mA in fixed 20 s intervals. The stimulation threshold was defined as the lowest pacing current resulting in slice contraction without any skipped beats. The refractory period was tested by applying regular stimuli with an interpulse interval of 2000 ms, followed by a secondary stimulus. The interval between the two stimuli decreased overtime from 960 ms to 80 ms, and the longest interval resulting in the absence of two contractions was defined as the refractory period. The force-frequency relationship was measured by pacing the slices at increasing frequencies (0.2, 0.33, 0.5, 0.75, 1, 1.33, 1.66, 2, 2.5, 3, 3.5 and 4 Hz). The contraction force developed at each frequency was measured and reported when 1) slices managed to follow the pacing frequency and 2) the amplitude difference between contractions during the same pacing frequency was not superior to 50%. To ensure the reliability of the quantifications, failure to fulfil any of the previous requirements resulted in the exclusion of the data obtained at that frequency. Post-pause potentiation was measured by introducing 3, 12 or 30 s pauses during regular 0.5 Hz (50 mA, biphasic, 3 ms) pulses. The contraction force of the 9 contractions prior to the pause was averaged and compared to the contraction force of the first contraction after the pause. Between pauses, slices were paced for 30 s using the regular pacing strategy. For all analyses, data is presented for days 0, 1, 6, and 10. Days 6 and 10 may deviate one day due to 48- and 72-hour media change intervals, ensuring data reflects periods without recent media exchange to avoid effects from fresh media. Contraction force was normalized to day 1 and absolute forces are displayed in Figure S1A. LabChart 8 Reader application from AD Instruments was used to conduct blinded data analysis.

**Optical calcium transient assessment**

Immediately after LMS preparation and following 10 days of culture, LMS were incubated for 15 minutes with a 25 µM Rhod2AM (Invitrogen, Oregon, US) supplemented with 10 µM blebbistatin and 0.15% Pluronic F-127 (Sigma-Aldrich) in a modified Tyrode’s solution (1.8 mM CaCl_2_, 10 mM glucose, 10 mM HEPES, 4.5 mM KCl, 1 mM MgCl_2_ and 140 mM NaCl at pH 7.4). Following this, the LMS underwent a 15-minute de-esterification wash and were subsequently transferred to an in-house designed recording chamber. The modified Tyrode’s solution was continuously perfused and heated to 37°C while being superfused with oxygen. Field stimulation was applied (Stimulator CS, Hugo Sachs Elektronik, Germany) with a 10 ms biphasic pulse at varying frequencies (0.5, 1, 2, 3 Hz, 30 seconds recordings of each). A custom-built microscope (Cairn Research, Faversham, UK) equipped with a macro-objective (MVPLAPO 1X, OLYMPUS, Tokyo, Japan) recorded the fluorescent signal. White light was filtered using a 545/30 nm excitation filter and directed onto the LMS with a 580-nm dichroic mirror. The resulting fluorescent signal was captured using a 605/55 nm long-pass emission filter by a high-speed camera (Andor Zyla 5.5.CL3, Oxford Instruments, Abingdon, UK). Subsequent data analysis was performed using Fiji and a custom-written Matlab script, Peaks (DOI:10.17605/OSF.IO/86UFE). The amplitudes are reported relative to the diastolic calcium levels. Following optical assessment of calcium handling, the tissue slices were either fixed in a 4% formaldehyde solution or snap-frozen for subsequent analysis.

**Histological analysis of LMS**

For immunofluorescent staining, paraffin-embedded myocardial slices were cut longitudinally to a thickness of 4 μm. First, sections were deparaffinized, subjected to antigen retrieval using a citrate buffer at pH 6.0, and then permeabilized using 0.1% Triton-X-100 for 5 minutes. Next, sections were blocked using 5% BSA for 1 hour at room temperature and incubated overnight at 4°C with the primary antibodies: Troponin I, (PA1-86820, ThermoFisher, 1:100, 10 μg/ml), and Connexin-43 (610062, BD Biosciences, 1:50, 5 μg/ml) or ATP5a (ab14748, Abcam 1:100, 10 μg/ml). After washing with 0.5% Tween-20, sections were incubated for 1 hour at room temperature with DAPI (D1306, ThermoFisher, 1:2000, 0.5 μg/ml) and secondary antibodies: Alexa-Fluor^TM^ 488 Donkey anti-Goat IgG (A-11055, ThermoFisher, 1:1000, 2 μg/ml) and Alexa-Fluor^TM^ 555 Donkey anti-Mouse IgG (A-31570, ThermoFisher, 1:1000, 2 μg/ml). Finally, the stained sections were washed and mounted with a coverslip using Mowiol 4-88 (Sigma-Aldrich). Image acquisition was conducted using a Leica SP8 confocal microscope. Image assembly, merging, and contrast/brightness adjustments were performed in Fiji 2.14.0/1.54i software.

**RNA sequencing**

RNA was isolated from snap-frozen LMS by bead-beating, followed by a TriPure isolation (Roche Diagnostics, Mannheim, Germany) according to the manufacturer’s instructions. RNA concentration and quality were assessed using the Agilent Bioanalyzer 4150 (Agilent). RNA samples underwent poly-A RNA library construction on the DNBseq^TM^-T7 and were sequenced on an Illumina NovaSeq platform, generating 20 million reads per sample (maximum length of PE150). The sequenced reads were then demultiplexed and aligned to the human cDNA reference (Ensembl v84) using the BWA with the following setting: ‘bwa aln’, ‘bwa sampe’ -n 100 -N 100. Differential gene enrichment expression between Day 0 and Day 10±1 was performed using DESeq2 (v1.38.3). The top 500 most differentially expressed genes were used for principal component analysis. The highest correlating pathways per category were identified, while the remaining pathways are in Supplemental Tables S1 and S2. Pathway analysis using ClusterProfiler (v4.6.2) identified significant enrichment for pathways with a false discovery rate-adjusted p-value below 0.1. Significantly enriched pathways of metabolism were assessed using EnhancedVolcano (v1.22.0). Expression levels of genes of interest were assessed using the pheatmap R package (v1.0.12).

**Single-cell RNA sequence lookup**

Upregulated genes were mapped to single cells using a publicly available single-cell dataset of the left ventricle from donor hearts [3] using Seurat (v5.1.0). A Venn diagram including the genes identified as cell-type enriched (in a comparison of one vs. all, p-value<0.05) is present in Supplemental Figure S2. The enriched pathways per cell type are provided in Supplemental Tables S3-S6.

**Conditioned medium analysis**

LMS’ conditioned medium was spun down at 600 x g to remove cellular debris. High-sensitivity troponin I, glucose, and lactate levels were measured using Atellica CH Analyzer (Siemens Healthineers, Erlangen, Germany) with a chemiluminescence immunoassay, employing High-Sensitivity Troponin I, Glucose hexokinase_3 and Lactacte_2 kits respectively. The levels were determined by comparing the fresh medium and the conditioned medium.

**References**

[1] C. Fischer, H. Milting, E. Fein, E. Reiser, K. Lu, T. Seidel, et al. Long-term functional and structural preservation of precision-cut human myocardium under continuous electromechanical stimulation in vitro. Nature communications 2019; 10 (1): 117.

[2] S.A. Watson, J. Duff, I. Bardi, M. Zabielska, S.S. Atanur, R.J. Jabbour, et al. Biomimetic electromechanical stimulation to maintain adult myocardial slices in vitro. Nature communications 2019; 10 (1): 2168.

[3] N.R. Tucker, M. Chaffin, S.J. Fleming, A.W. Hall, V.A. Parsons, K.C. Bedi, Jr., et al. Transcriptional and Cellular Diversity of the Human Heart. Circulation 2020; 142 (5): 466-482.

**Supplemental Figures:**

**
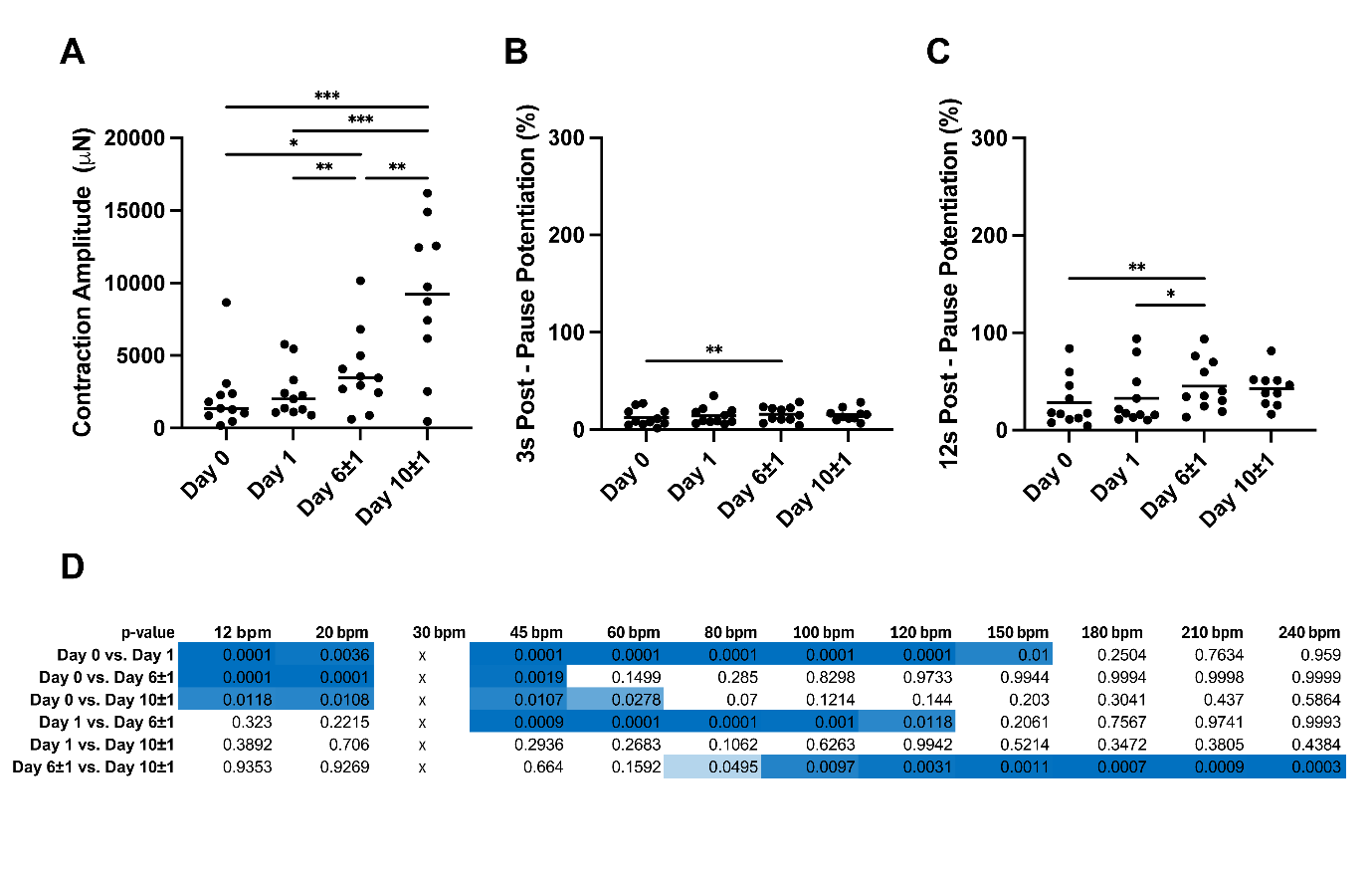
**

**Figure S1: Functional remodeling of LMS during culture.** A) Absolute contraction force developed by LMS increases over culture. Post-pause potentiation at 3s (B) and 12s (C) increases during culture. D) Tukey's multiple comparisons of force-frequency during culture of LMS (A-D: Day 0 N=11, n=126; Day 1 N=11, n=61; Day 6±1 N=11, n=49; N=10, n=22). N refers to the number of unique patients and n refers to individual LMS. Statistical analyses were performed using a mixed-effects model accounting for repeated measures within subjects, followed by Tukey’s multiple comparisons test. Significance was set at p < 0.05.

**
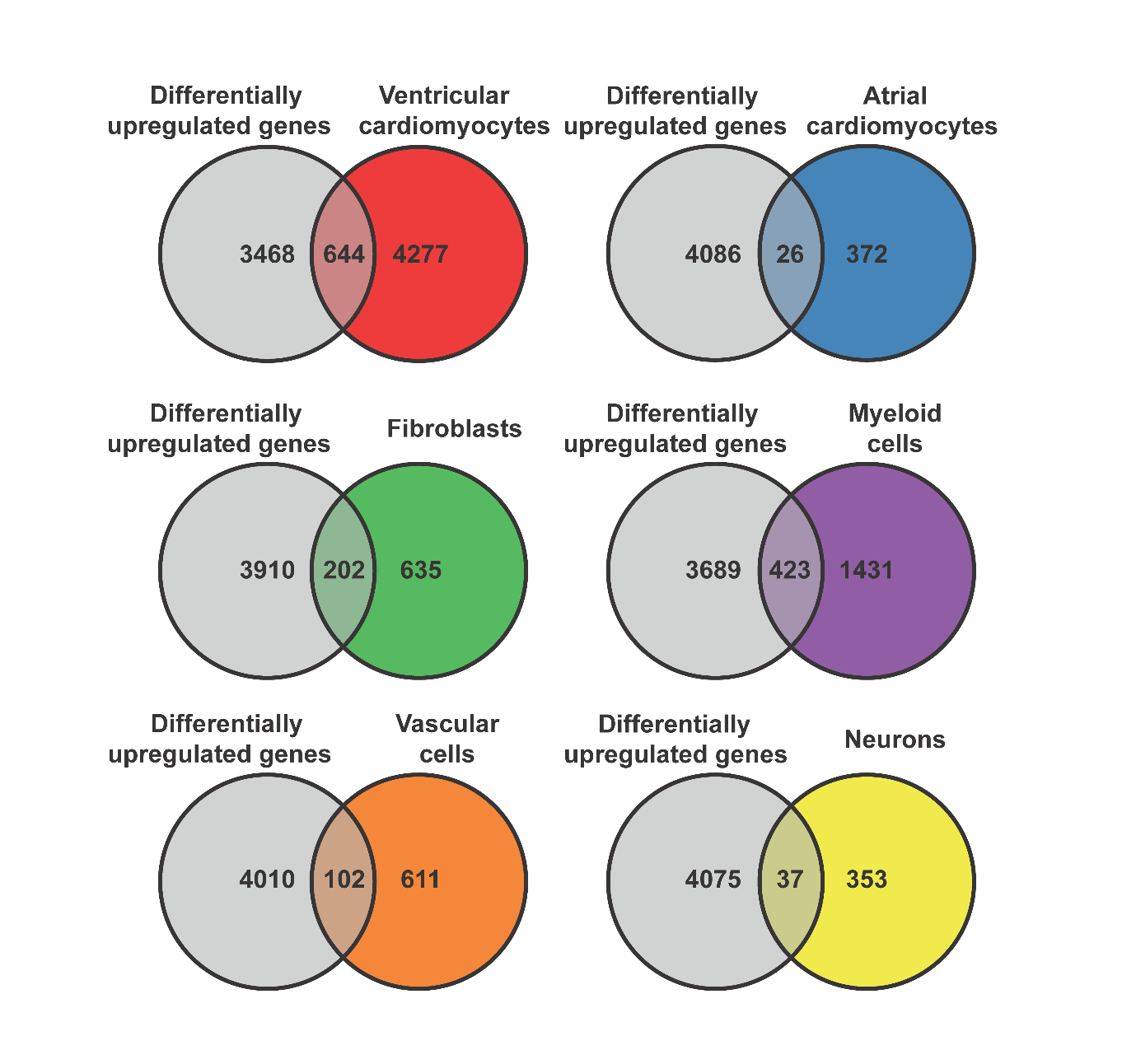
**

**Figure S2: Venn diagram illustrating the overlap of upregulated genes with specific cell markers in** **the heart.** The chart highlights the number of markers associated with ventricular and atrial cardiomyocytes, fibroblasts, vascular cells, myeloid cells, and neurons, alongside their enrichment in upregulated genes between day 0 and day 10 ± 1 (N=6 unique patients, n=6 individual LMS).
